# Decision-making in surgical neonatal necrotizing enterocolitis

**DOI:** 10.4103/0971-9261.59613

**Published:** 2009

**Authors:** Viroj Wiwanitkit

**Affiliations:** Wiwanitkit House, Bangkhae, Bangkok, Thailand 10160

Sir,

I read the recent publication by Parikh *et al*. with great interest.[1] Parikh *et al*. reached to the conclusion that “In the present study, neonate with persistently low pH, higher base deficit and presentation with shock predicted need for laparotomy in drain managed patients as well as chances of survival.[1]” This suggestion should be carefully considered. There are some points to be noted. First, the retrospective nature of this work might include some errors and bias in recorded data. Second, the number of subjects in this work might be too few to reach the statistical significance. Third, the other important underlying conditions of all neonates including the nutritional status, which is an important indicator in surgery, are not well documented. Indeed, there are some papers on the surgical management of the neonatal necrotizing enterocolitis. Of interest, Guner *et al*. noted that “Neonates treated surgically had a greater mortality rate compared with ones treated nonsurgically.[2]” These information should also be kept in mind when making a decision on the surgical management for the neonates with necrotizing enterocolitis.
